# Use of vitamin supplements and risk of total cancer and cardiovascular disease among the Japanese general population: A population-based survey

**DOI:** 10.1186/1471-2458-11-540

**Published:** 2011-07-08

**Authors:** Azusa Hara, Shizuka Sasazuki, Manami Inoue, Taichi Shimazu, Motoki Iwasaki, Norie Sawada, Taiki Yamaji, Junko Ishihara, Hiroyasu Iso, Shoichiro Tsugane

**Affiliations:** 1 Epidemiology and Prevention Division, Research Center for Cancer Prevention and Screening, National Cancer Center, 5-1-1 Tsukiji, Chuo-ku, Tokyo 104-0045, Japan; 2 Department of Nutrition, Junior College of Tokyo University of Agriculture, 1-1-1 Sakuragaoka, Setagaya-ku, Tokyo 156-8502, Japan; 3 Public Health, Department of Social and Environmental Medicine, Graduate School of Medicine, Osaka University, 2-2 Yamadaoka, Suita-shi, Osaka 565-0871, Japan

## Abstract

**Background:**

Despite the popular use of vitamin supplements and several prospective cohort studies investigating their effect on cancer incidence and cardiovascular disease (CVD), scientific data supporting their benefits remain controversial. Inconsistent results may be partly explained by the fact that use of supplements is an inconsistent behavior in individuals. We examined whether vitamin supplement use patterns affect cancer and CVD risk in a population-based cohort study in Japan.

**Methods:**

A total of 28,903 men and 33,726 women in the Japan Public Health Center-based Prospective Study cohort, who answered questions about vitamin supplement use in the first survey from 1990-1994 and the second survey from 1995-1998, were categorized into four groups (never use, past use, recent use, and consistent use) and followed to the end of 2006 for cancer and 2005 for CVD. Sex-specific hazard ratios (HRs) and 95% confidence intervals (95% CIs) were used to describe the relative risks of cancer and CVD associated with vitamin supplement use.

**Results:**

During follow-up, 4501 cancer and 1858 CVD cases were identified. Multivariate adjusted analysis revealed no association of any pattern of vitamin supplement use with the risk of cancer and CVD in men. In women, consistent use was associated with lower risk of CVD (HR 0.60, 95% CI 0.41-0.89), whereas past (HR 1.17, 95% CI 1.02-1.33) and recent use (HR 1.24, 95% CI 1.01-1.52) were associated with higher risk of cancer.

**Conclusions:**

To our knowledge, this is the first prospective cohort study to examine simultaneously the associations between vitamin supplement use patterns and risk of cancer and CVD. This prospective cohort study demonstrated that vitamin supplement use has little effect on the risk of cancer or CVD in men. In women, however, consistent vitamin supplement use might reduce the risk of CVD. Elevated risk of cancer associated with past and recent use of vitamin supplements in women may be partly explained by preexisting diseases or unhealthy background, but we could not totally control for this in our study.

## Background

Despite the popular use of vitamin supplements, the strong consumer belief is that they prevent chronic diseases such as cancer and cardiovascular disease (CVD) [1,2], but results from randomized controlled trials are mixed [3-10]. Most randomized controlled trials show little support of a preventive effect of vitamin supplement use and even increased risk [6,7] for cancer and CVD incidence and mortality, with some exceptions [8-10]. However, data from randomized controlled trials suffer from concerns about overreliance on secondary rather than primary prevention, insufficient intervention and follow-up periods, particularly regarding the incidence of cancer, inappropriate supplement doses, and unsuitable cohorts for testing the hypothesis. Therefore, studies for the effects of long-term, low doses of several agents in the general population are needed. Despite several prospective cohort studies investigating their effect on cancer incidence (all site [11,12], colorectal [13-15], breast [16,17], lung [18,19], prostate [20-23], non-Hodgkin lymphoma [24]), and CVD incidence [11,25-29], scientific data supporting their benefits remain controversial. Inconsistent results may be partly explained by the fact that use of supplements is an inconsistent behavior in individuals [13,30]. Individuals with a favorable lifestyle and healthy diet are more likely to use vitamin supplementation consistently [30]. Some studies have found reduced risk for incidence and mortality of cancer and CVD associated with a long duration of vitamin supplement use [13,14,27,28,31-34]; however, consistent use of vitamin supplements could not be clearly determined by using a single time-point survey at baseline.

It is also important to note that the use of vitamin supplements is often associated with healthy lifestyle factors or with specific health issues, such as hypertension and cancer, that may increase or decrease vitamin supplement use [35,36]. In Japan, a few cross-sectional studies reported that the prevalence of vitamin supplement use was approximately 10% to 30% of the study population and that vitamin supplement use was associated with several factors broadly characterized by health consciousness and conversely by poor health [37-39]. However, all prospective studies have been conducted in Western populations (United States [11-15,17-29,31-34] and European countries [16,19,25]). No data have been reported for prospective cohort studies in Asian general populations, although there are some randomized clinical trials [8,40].

Therefore, we examined the association between vitamin supplement use and the risk of cancer and CVD in a population-based prospective cohort study in Japan. Participants in this cohort reported vitamin supplement use at two time points, which enabled us to examine the impact of the pattern of use on the risk of cancer and CVD.

## Methods

The Japan Public Health Center (JPHC)-Based Prospective Study was started in 1990 for cohort I and in 1993-1994 for cohort II. All subjects were Japanese inhabitants registered at 11 public health center areas and were aged 40-69 years at the time of their first survey. Details of the study design have been described previously [41]. In the present study, the subjects from two public health center areas (Katsushika in Tokyo prefecture and Suita in Osaka prefecture) were excluded because the incidence data for cancer or CVD were not available and the selection of subjects differed from that in other public health center areas. Of 116,896 people in nine public health areas, 95,405 (82%) individuals responded to the first survey. We excluded 1168 persons who were not Japanese, who had died or moved out of a study area, or who were lost to follow-up before the starting point. This left 94,237 eligible subjects. In 1995 and 1998, the second survey was conducted; 79,809 subjects replied (85%; 36,783 men and 43,026 women) and were included in the present study. The institutional review board of the National Cancer Center, Tokyo, Japan, approved the study.

The status of vitamin supplement use was defined by the responses in the two surveys and was classified into the following four categories of use: (1) never, no vitamin supplement use in either the first or second survey; (2) past, vitamin supplement use only in the first survey; (3) recent, vitamin supplement use only in the second survey; and (4) consistent, vitamin supplement use in both surveys. In the first survey, cohort I and cohort II subjects were asked how frequently they used vitamin supplements. Those who reported use on >1 day/week were asked about the type of vitamin supplements. Use of vitamin supplements in the first survey was defined as subjects who used them at least 1 day/week. No information was collected on brand name or duration of vitamin supplement use. In the second survey for cohorts I and II, general use of any vitamin supplements more than once a week and use of specific vitamin supplements were examined. The brand names of vitamin supplements used were requested, and 81.7% provided this information. We used re-categorized self-reported categories of vitamin supplements based on the definition in the Women's Healthy Eating and Living Study [42] to improve sensitivity in identifying supplement use [43]. Details of the assessment of self-reported vitamin supplement use have been described previously, and use of vitamin supplements in the second survey was defined as subjects who used at least one type of vitamin supplement ≥ 1 week for ≥ 1 year [37,43].

We followed subjects from the second survey until December 31, 2006, for cancer and until December 31, 2005, for CVD. We identified changes in residence status and survival annually through the residential registry in each area or, for those who had moved out of the area, by using the municipal office of the area to which they had moved. Residency registration and death registration are required by the Basic Residential Register Law and Family Registry Law, respectively, and the registries are thought to be complete. During the follow-up period, 8060 subjects (10.1%) died, 2106 (2.6%) moved out of the study areas, and 249 persons (0.31%) were lost to follow-up between the second survey and December 31, 2006.

The occurrence of cancer was identified by active patients' notification from major local hospitals in the study area, that is, the extraction of clinical information from medical records into cohort-specific registration forms in either local major hospitals, which care for most of the patients with cancer or CVD (up to 80%) in some areas, by physicians in the hospital or physicians in the public health center [44], and from data linkage with population-based cancer registries, with permission from each of the local governments responsible for the cancer registries. Cases of cancer were coded according to the *International Classification of Disease for Oncology*, third edition, of the World Health Organization [45]. In our cancer registry system, the proportion of cases for which information was available from death certificates only was 4.4%. For the present analysis, the earliest date of diagnosis was used in cases with multiple cancer diagnoses at different times. Diagnosis of myocardial infarction according to the criteria of the Monitoring Trends and Determinants of Cardiovascular Disease (MONICA) project [46] and diagnosis of stroke according to the criteria of the National Survey of Stroke [47] were confirmed for all cases by computer tomographic scan, magnetic resonance imaging, or both as recorded in the medical record and reviewed by hospital or public health center physicians in each registered major local hospital in each public health center area [48,49]. CVD cases with a death certificate or by self-report only, without confirmation by medical records, were treated as non-CVD cases. CVD was defined as myocardial infarction or stroke, whichever occurred first. Among the 79,809 subjects, we confirmed 5932 cases of newly diagnosed cancer by December 31, 2006, and 3218 cases of CVD by December 31, 2005. Participants with both cancer and CVD were included in both analyses.

From the 79,809 respondents, we excluded subjects with a history of cancer or CVD (*n *= 5809) and those who did not have information on their vitamin supplement use in both surveys (*n *= 11,371). Subjects with a history of cancer or CVD were defined as diagnosed with cancer or CVD before the starting point or from self-reports in the surveys. For the final analysis, 62,629 subjects (28,903 men and 33,726 women) remained, including 4501 with cancer and 1858 with CVD. We calculated person-years of follow-up for each subject from the starting point to the date of diagnosis, date of emigration from the study area, date of death, or end of the follow-up (December 31, 2006 for the cancer analysis and December 31, 2005 for the CVD analysis), whichever came first. We censored subjects lost to follow-up at the last confirmed date they were present in the study area. A total of 597,281 person-years were accrued for the cancer analysis and 547,983 for the CVD analysis. Sex-specific hazard ratios (HRs) and 95% confidence intervals (95% CIs) were used to describe the relative risks of total cancer and CVD associated with use of vitamin supplements. The Cox proportional hazards model was used to control for potential confounding factors, which were either known or suspected from previous studies as risk factors for cancer and CVD. All covariates were based on information from the second survey. We conducted the initial analyses by adjusting for age at the starting point (5-year groups) and study area (nine public health center areas). In the multivariate model, we further adjusted for smoking status (never, former, <20, 20-29, 30-39, and ≥ 40 pack-years for men, and never, former, <20, and ≥ 20 pack-years for women), alcohol consumption (none, <150, 150-299, 300-449, and ≥ 450 g ethanol/week for men, and none, <150, and ≥ 150 g ethanol/week for women), body mass index ([BMI] <19, 19-20.9, 21-22.9, 23-24.9, 25-26.9, 27-29.9, and ≥ 30 kg/m^2^), occupation (farming, forestry, and fishing; employee and professional; housewife; self-employed; unemployed; other occupations; and combination [≥ 2 occupations across those groups]), quartile of physical activity in metabolic equivalent task-hours/day, total energy intake, energy-adjusted green vegetable intake, current medication status (hypertension, hyperlipidemia, or diabetes mellitus), and screening examination (blood pressure measurement, biochemical examination, electrocardiogram, fundus examination, chest radiograph, sputum cytology, gastric photofluorography, gastrointestinal endoscopy, fecal occult blood test, barium enema, or colonoscopy for men and women, and mammography or Papanicolaou smear for women), which were reported in a questionnaire in the second survey. As for current medication status and screening examination, if a subject replied "yes" to at least one medication or examination, we regarded the subject as using medication or taking the examination, respectively. The second survey included a food-frequency questionnaire consisting of 138 food items with standard portions/units and nine frequency categories, which were developed to estimate dietary intake [50] and validated for estimations of various nutrients and food groups [51-54]. A residual model was used for energy adjustment of green vegetable consumption, vitamin B_2_, vitamin B_6_, vitamin B_12_, folate, α-tocopherol, vitamin C, and vitamin D intake reported in food-frequency questionnaire [55]. Extreme values of BMI (<14 or ≥ 40 kg/m^2^) and total energy intake (lower and upper 2.5 percentiles) were treated as missing values. Statistical significance was assumed at *P *< 0.05. All statistical analyses were performed using SAS software, version 9.1 (SAS Institute, Cary, NC, USA).

## Results

Of the participants included in this analysis, 49,060 subjects (78.3%) reported no vitamin supplement use, 7833 subjects (12.5%) reported only past vitamin supplement use (in the first survey), 2593 subjects (4.2%) reported only recent vitamin supplement use (in the second survey), and 3143 subjects (5.0%) reported past and recent vitamin supplement use. Among subjects who used vitamin supplements and reported the brand name in the second survey, the most common vitamin supplement was B vitamins for men and women (multivitamin: 474 subjects [25.8%] and 566 subjects [19.6%]; antioxidants: 30 subjects [1.6%] and 126 subjects [4.4%]; vitamin A: 65 subjects [3.5%] and 144 subjects [5.0%]; B vitamins: 797 subjects [43.5%] and 883 subjects [30.6%]; vitamin C: 299 subjects [16.3%] and 656 subjects [22.7%]; vitamin E: 295 subjects [16.1%] and 843 subjects [29.2%]; other vitamins: 219 subjects [11.9%] and 443 subjects [15.3%], respectively).

Table 1 shows the baseline characteristics of the study subjects according to vitamin supplement use pattern in men and women separately. Individuals with past use and consistent use of vitamin supplements were significantly older for both sexes. Men who had never used supplements were thought to have lower health consciousness due to higher proportions with a BMI ≥ 25 kg/m^2^, a greater likelihood of being a smoker or regular drinker, less information on their disease history (angina, diabetes, colonic polyp, and hepatitis), fewer screening examinations, and less consumption of soy foods and fruits compared with other men. Significantly higher proportions of men with consistent supplement use took more medications (hyperlipidemia and diabetes), were more likely to have disease histories (angina, diabetes, duodenal ulcer, colonic polyp, and hepatitis), and may have higher health consciousness suggested by lower BMI, less regular drinking, more screening examinations, and higher consumption of fruits. Men with past supplement use also had a significantly higher proportion of antihypertensive medication use. Men with recent use also tended to have a healthy lifestyle and significantly lower proportions were smokers or taking diabetic medication. Women who had never used supplements were likely to have a healthier lifestyle, with significantly lower proportions being smokers or regular drinkers than other women. Women with recent or consistent use were also basically health conscious, having a lower BMI and a higher proportion of screening examinations, despite there being a significantly higher proportion of regular drinkers. Individuals with consistent use also consumed significantly larger amounts of fruits, folate, and vitamin C. They also tended to have significantly higher proportions of medication use (hypertension and hyperlipidemia) and history of diseases such as gastric and colonic polyps than those who never used supplements. Women with recent use were also more likely to have a history of gastric and colonic polyps, despite their younger age, and had a significantly higher proportion of medication use except for hypertension, hyperlipidemia, and diabetes. Women with past use tended to have an unhealthy lifestyle, including a higher BMI and a greater likelihood of smoking and medication use (hypertension and diabetes).

**Table 1:** Population characteristics according to supplement use categories, Japan Public Health Center-based Prospective Study

		**Men (*n *= 28,903**)					Women (*n *= 33,726)				
			
		Never use	Past use	Recent use	Consistent use	*P*	Never use	Past use	Recent use	Consistent use	*P*
No. (%)		23,535 (81.4)	3161 (10.9)	1026 (3.6)	1181 (4.1)		25,525 (75.7)	4672 (13.9)	1567 (4.6)	1962 (5.8)	
Age in years (mean (SE*))		55.7 (0.05)	57.9 (0.1)	57.1 (0.2)	58.2 (0.2)	<.0001	56.1 (0.05)	57.9 (0.1)	56.7 (0.2)	57.8 (0.2)	<.0001
Body mass index ≥ 25 kg/m^2 ^(%)		29.1	26.9	27.1	25.9	0.0049	29.0	31.0	26.3	23.6	<.0001
Smoking status (%)	Former smoker	16.6	18.6	19.5	21.3	<.0001	0.7	1.2	1.7	1.4	<.0001
	Current smoker	47.8	44.8	41.4	42.2		4.3	5.7	4.7	5.2	
Regular drinker, ≥ 150 g ethanol/wk (%)		50.5	46.1	46.8	45.5	<.0001	2.4	2.6	3.1	3.6	0.002
Mean MET*-hours/d	(mean (SE))	33.1 (0.05)	32.9 (0.1)	33.3 (0.2)	32.7 (0.2)	0.06	32.2 (0.04)	31.9 (0.09)	32.3 (0.2)	32.0 (0.1)	0.003
Medication (%)	Hypertension	16.7	21.9	17.0	21.5	<.0001	17.8	24.4	18.1	20.0	<.0001
	Hyperlipidemia	3.0	4.3	3.9	4.9	<.0001	6.0	7.8	7.5	9.3	<.0001
	Diabetes	3.2	4.2	2.8	4.5	0.001	2.0	2.8	2.2	1.5	0.0005
	Others	12.2	17.1	15.4	15.1	<.0001	10.9	13.1	16.3	15.9	<.0001
History (%)	Angina	0.9	1.3	1.1	1.6	0.03	1.0	1.2	1.5	1.2	0.2
	Diabetes	5.8	6.6	5.8	7.5	0.04	3.0	3.4	3.3	2.5	0.2
	Gastric ulcer	5.0	5.4	5.0	4.9	0.8	2.1	2.3	2.6	2.9	0.07
	Duodenal ulcer	2.5	2.0	1.9	3.5	0.02	1.2	1.2	1.4	1.3	0.9
	Gastric polyp	2.2	2.3	2.6	3.3	0.09	2.6	2.4	3.3	3.7	0.004
	Colonic polyp	4.1	4.3	4.9	5.8	0.03	1.8	1.7	2.0	3.3	<.0001
	Hepatitis	1.7	1.9	2.3	3.2	0.0004	0.7	0.9	1.2	1.2	0.005
Screening examination (%)		83.3	84.0	87.0	88.0	<.0001	86.0	86.3	88.8	89.9	<.0001
Total energy intake (kcal/day) (mean (SE))		2206 (4.2)	2200 (12.1)	2222 (20.4)	2228 (18.1)	0.5	1886 (3.6)	1879 (8.6)	1938 (14.5)	1925 (12.9)	0.0001
Energy-adjusted	Salt intake (g/d)	12.2 (0.03)	12.3 (0.07)	12.4 (0.1)	12.3 (0.1)	0.5	12.0 (0.08)	11.9 (0.05)	11.7 (0.09)	11.7 (0.08)	0.5
food intake	Soy food (g/d)	86 (0.5)	93 (1.6)	89 (2.3)	89 (1.8)	0.0001	86 (0.5)	94 (1.2)	86 (1.6)	89 (1.6)	<.0001
(mean (SE))	Green vegetables (g/d)	38 (0.2)	38 (0.6)	41 (1.2)	40 (1.0)	0.01	48 (0.2)	47 (0.6)	50 (1.1)	49 (0.8)	0.055
	Fruits (g/d)	172 (1.0)	187 (2.9)	189 (4.8)	204 (4.7)	<.0001	239 (1.1)	242 (2.5)	242 (4.0)	254 (3.9)	0.001
	Fish (g/d)	91 (0.4)	91 (1.0)	92 (1.8)	92 (1.5)	0.7	87 (0.3)	86 (0.7)	86 (1.2)	86 (1.0)	0.4
	Red meat (g/d)	52 (0.3)	51 (0.7)	53 (1.3)	51 (1.0)	0.5	46 (0.2)	48 (0.5)	45 (0.8)	46 (0.7)	0.0009
Energy-adjusted	α-tocopherol (mg/d)	6.6 (0.02)	6.8 (0.05)	7.1 (0.08)	7.1 (0.07)	<.0001	7.3 (0.01)	7.5 (0.03)	7.5 (0.05)	7.6 (0.05)	<.0001
nutrition intake	Vitamin B_1 _(mg/d)	1.05 (0.003)	1.10 (0.008)	1.08 (0.01)	1.11 (0.01)	<.0001	1.08 (0.002)	1.12 (0.006)	1.11 (0.009)	1.11 (0.008)	<.0001
(mean (SE))	Vitamin B_2 _(mg/d)	1.41 (0.003)	1.45 (0.009)	1.50 (0.02)	1.52 (0.01)	<.0001	1.43 (0.003)	1.46 (0.007)	1.51 (0.01)	1.54 (0.01)	<.0001
	Niacin (mg/d)	20.1 (0.04)	20.1 (0.1)	20.6 (0.2)	20.4 (0.1)	0.002	18.1 (0.03)	18.1 (0.07)	18.2 (0.1)	18.3 (0.09)	0.08
	Vitamin B_6 _(mg/d)	1.56 (0.002)	1.58 (0.006)	1.60 (0.01)	1.60 (0.01)	<.0001	1.46 (0.002)	1.47 (0.004)	1.48 (0.008)	1.49 (0.007)	<.0001
	Vitamin B_12 _(μ g/d)	9.1 (0.03)	9.2 (0.09)	9.3 (0.1)	9.4 (0.1)	0.02	8.6 (0.03)	8.7 (0.06)	8.7 (0.1)	8.6 (0.09)	0.2
	Folate (μ g/d)	377 (0.9)	385 (2.7)	401 (4.9)	399 (4.1)	<.0001	409 (0.9)	413 (2.2)	422 (3.8)	426 (3.1)	<.0001
	Pantothenic acid (mg/d)	6.7 (0.01)	6.8 (0.03)	6.9 (0.05)	7.1 (0.05)	<.0001	6.6 (0.008)	6.7 (0.02)	6.8 (0.03)	6.9 (0.03)	<.0001
	Vitamin C (mg/d)	118 (0.4)	122 (1.2)	128 (2.2)	130 (1.9)	<.0001	151 (0.5)	149 (1.1)	154 (1.7)	158 (1.6)	<.0001
	Vitamin D (mg/d)	10.1 (0.04)	10.0 (0.1)	10.4 (0.2)	10.3 (0.2)	0.2	10.0 (0.04)	9.9 (0.09)	9.9 (0.1)	9.9 (0.1)	0.4

Never use, neither past nor recent use; Past use, past use but not recent use; Recent use, recent use but not past use; Consistent use, both past and recent use.

*SE, standard error; MET, metabolic equivalent task.

Associations of vitamin supplement use pattern and total cancer and CVD risk in men and women are shown separately in Table 2. In men, no significant association was found between any pattern of vitamin supplement use and the risk of total cancer and CVD in age- and study area-adjusted and multivariate-adjusted models. No significant association was found between any specific vitamin supplement use in the second survey and total cancer and CVD. For women, however, a statistically significant increase in the risk of total cancer occurrence was observed in those with past and recent vitamin supplement use compared with those who never used supplements; the HR of developing cancer (95% CI) for past use and recent use was 1.17 (1.02-1.33) and 1.24 (1.01-1.52), respectively. When we performed separate analyses for major site-specific cancers, the HR of recent use in women was especially high for stomach cancer (HR 2.15, 95% CI 1.39-3.34). We also observed a nonsignificant but moderately increased risk of liver and pancreatic cancer with past supplement use in women (liver cancer: HR 1.61, 95% CI 0.95-2.74; pancreatic cancer: HR 1.67, 95% CI 0.94-2.97). When we estimated the HR after excluding women diagnosed as having cancer within 5 years of baseline, similar trends were observed, although the association for cancer with recent use was not significant and with past use remained significant. In the second survey, vitamin C supplements specifically and antioxidant supplementation, including two or more of β-carotene, vitamin C, vitamin E, and selenium [42], were significantly associated with an increased risk of total cancer; compared with the subjects with no vitamin supplement use, the HR and 95% CI of vitamin C supplement and antioxidant supplement use were 1.38 (1.03-1.87) and 1.83 (1.01-3.31), respectively. In contrast, we observed a statistically significant reduced risk for CVD with consistent vitamin supplement use for women (HR 0.60, 95% CI 0.41-0.89). When we performed separate analyses for coronary heart disease, hemorrhagic stroke, or ischemic brain infarction, decreased risk was observed for ischemic brain infarction with statistical significance with consistent use (coronary heart disease: HR 0.19, 95% CI 0.03-1.34; hemorrhagic stroke: HR 0.61, 95% CI 0.29-1.31; ischemic brain infarction: HR 0.52, 95% CI 0.28-0.98). HR estimates after excluding women diagnosed with CVD within 5 years of baseline showed a similar trend to estimates using all cases, although they were not statistically significant.

**Table 2:** Hazard ratios for total cancer and cardiovascular disease according to supplement use categories

			Total					Excluding cases within 5 years		
				
		Person-years	No. of cases	HR*1 (95% CI*)	*P*	HR2 (95% CI)	*P*	No. of cases	HR2 (95% CI)	*P*
Men										
			*Total cancer*							
	Never use	220,948	2152	1.00 (reference)		1.00 (reference)		1210	1.00 (reference)	
	Past use	28,863	324	0.98 (0.87-1.10)	0.8	0.98 (0.87-1.10)	0.8	167	0.95 (0.80-1.11)	0.5
	Recent use	9603	102	1.00 (0.82-1.22)	0.97	1.01 (0.83-1.23)	0.9	59	1.05 (0.80-1.36)	0.7
	Consistent use	10,863	139	1.11 (0.94-1.32)	0.2	1.10 (0.93-1.31)	0.3	75	1.13 (0.89-1.43)	0.3
			*Cardiovascular disease*							
	Never use	203,013	934	1.00 (reference)		1.00 (reference)		490	1.00 (reference)	
	Past use	26,639	125	0.91 (0.75-1.09)	0.3	0.89 (0.73-1.07)	0.2	61	0.86 (0.66-1.12)	0.3
	Recent use	8889	31	0.71 (0.50-1.02)	0.06	0.72 (0.51-1.04)	0.08	15	0.66 (0.39-1.10)	0.1
	Consistent use	10,059	53	1.03 (0.78-1.36)	0.8	1.02 (0.77-1.35)	0.9	28	1.04 (0.71-1.53)	0.8
										
Women										
			*Total cancer*							
	Never use	248,659	1299	1.00 (reference)		1.00 (reference)		698	1.00 (reference)	
	Past use	44,237	287	1.19 (1.04-1.35)	0.01	1.17 (1.02-1.33)	0.02	157	1.21 (1.01-1.44)	0.04
	Recent use	15,217	101	1.25 (1.02-1.53)	0.03	1.24 (1.01-1.52)	0.04	56	1.26 (0.96-1.66)	0.1
	Consistent use	18,892	97	0.94 (0.76-1.16)	0.6	0.92 (0.75-1.13)	0.4	47	0.82 (0.61-1.11)	0.2
			*Cardiovascular disease*							
	Never use	227,570	530	1.00 (reference)		1.00 (reference)		262	1.00 (reference)	
	Past use	40,586	116	1.11 (0.91-1.36)	0.3	1.08 (0.88-1.32)	0.5	63	1.24 (0.94-1.64)	0.1
	Recent use	13,918	43	1.30 (0.95-1.77)	0.1	1.32 (0.97-1.81)	0.08	20	1.26 (0.80-1.99)	0.3
	Consistent use	17,309	26	0.60 (0.40-0.89)	0.01	0.60 (0.41-0.89)	0.01	14	0.70 (0.41-1.21)	0.2

Never use, neither past nor recent use; Past use, past use but not recent use; Recent use, recent use but not past use; Consistent use, both past and recent use.

*HR, hazard ratio; CI, confidence interval.

HR1: Adjusted for age and public health center area.

HR2: Further adjusted for body mass index, smoking status, ethanol intake, occupation, daily total physical activity level, green vegetable intake, total energy intake, medication, and screening examination.

These statistically significant findings remained unchanged when we further adjusted dietary vitamin B_2_, B_6_, B_12_, folate, α-tocopherol, vitamin C, and vitamin D intake separately and simultaneously (data not shown).

Age, smoking status, alcohol intake, and dietary intake of vitamin B_2_, B_6_, B_12_, folate, α-tocopherol, vitamin C, and vitamin D did not significantly interact with any of the above results (for all interactions, *P *> 0.5).

## Discussion

In this prospective cohort study in an Asian population, we found that vitamin supplement use has little effect on the risk of total cancer or CVD in men. In women, however, past and recent use of vitamin supplements may be associated with higher risk of cancer, whereas consistent use may be associated with lower risk of CVD.

Several observational studies have examined the association between vitamin supplements and the risk of cancer and CVD incidence, but results have varied [11-29], partly because vitamin supplement use is an inconsistent behavior in individuals [13,30]. In our study, we found that only 4.1% of men and 5.8% of women continued to use vitamin supplements from the first to the second survey. Although some studies have found reduced incidence and mortality risk of cancer and CVD with a long duration of vitamin supplement use [13,14,27,28,31-34], to our knowledge, only limited data are available to clarify the consistency of vitamin supplement use over two surveys [13,31]. One prospective cohort study in the United States investigated consistency for vitamin supplement use through two surveys among 145,260 subjects, observing 797 incident cases of colorectal cancer, and found that multivitamin supplement use in the first survey and in both surveys was associated with reduced risk of colorectal cancer, whereas multivitamin supplement use in the second survey had no association with the disease [13]. Another study, in which 3490 deaths were observed among 11,178 study subjects in the United States, found that use of vitamin E supplements at two points within a relatively short period (baseline and study inception 3 years earlier) was associated with reduced risk of coronary heart disease mortality, whereas use at one point did not show significant association in multivariate analysis [31].

In the present study, the inverse associations for CVD, especially for ischemic brain infarction, was observed with consistent supplement use in women. It is known that homocysteine may promote atherogenesis by damaging the vascular matrix, increasing the proliferation of endothelial cells, and facilitating oxidative injury to vascular walls [56-58] and may be related to CVD [59,60]. Although several large trials of homocysteine-lowering B-vitamin therapy have all failed to demonstrate a reduction in coronary heart disease risk, some studies have shown possible evidence for stroke [9,10]. It has also been reported that B vitamins are important enzymatic cofactors in the synthesis of methionine from homocysteine and that a deficiency in any of them raises homocysteine concentrations in the blood [61,62]. In the present study, when we adjusted for several kinds of dietary B vitamins (vitamin B_2_, vitamin B_6_, vitamin B_12_, and folate), similar results were observed. Moreover, the most common vitamin supplement in the second survey was B vitamins in men and women in the present study (36.1% and 25.0%, respectively, among vitamin supplement users). Therefore, the inverse association between the consistent use of vitamin supplement and risk of CVD in women, especially ischemic brain infarction, might be caused by supplementation with B vitamins.

Alternatively, past and recent use of vitamin supplements was associated with higher risk of cancer in women. Women with past use tended to have unhealthy characteristics, such as a higher BMI, a greater likelihood of smoking, and medication use (hypertension and diabetes). Recent use in women may have been prompted by symptoms of ill health because women with recent use had a higher proportion of disease histories (e.g., gastric and colonic polyps) despite their younger age and had a significantly higher proportion of medication use except for hypertension, hyperlipidemia, and diabetes. Furthermore, the association of cancer with recent use was not significant when we estimated the HR after excluding women diagnosed as having cancer within 5 years of baseline, though that might be partly caused by the decreased number of cases. Elevated risk may be partly explained by characteristics of the women that were not measured or could not be controlled for in our study. Moreover, it might be partly caused by a pro-oxidant effect of supplementation with vitamin C [63-65], producing DNA damage and increasing the risk of cancer, because use of vitamin C in the second survey was associated with increased risk of total cancer among women. Furthermore, high-dose antioxidant supplementation might cause an increased risk of cancer among a high-risk group; in addition, two large, randomized clinical trials in which high doses of β-carotene were used, the Beta-Carotene And Retinol Efficacy Trial (CARET) in the United States and the Alpha-Tocopherol, Beta-Carotene Cancer Prevention (ATBC) trial in Finland, found that β-carotene, alone or in combination with vitamin E or retinyl palmitate, increased the incidence of lung cancers compared with placebo among high-risk groups, such as heavy smokers and those with a history of exposure to asbestos [6,7].

In the present study, vitamin supplement use was associated with the risk of total cancer or CVD in women but not in men. The characteristics of subjects with each vitamin supplement pattern were different between men and women, suggesting that these characteristics and unmeasured or residual confounders might cause the sex-based difference in results.

Our study has a potential limitation due to the differences in questionnaires regarding vitamin supplement use between the first and second surveys, and these differences might cause misclassification of vitamin supplement use prevalence, which was lower in the second survey than that in the first survey. Short-time vitamin supplement use of <1 year was regarded as vitamin supplement use in the first survey. In the second survey, re-categorized self-reported categories of vitamin supplementation were used to improve sensitivity in identifying vitamin supplement use [43] and vitamin supplement use was defined by vitamin supplements being taken ≥ 1 time/week for a year or longer. Information of duration was not available in the first survey. In addition, the possibility of selection bias needs to be considered when generalizing the present findings because 15% of the eligible subjects did not reply in the second survey. In our previous report, risks of mortality for all causes, all cancers, and CVD were higher among non-responders to the first survey compared with responders and elevated risk for cancer was observed only in the first 2 years of follow-up, whereas that for stroke was relatively stable for the entire period [66].

The strength of this study was its prospective design, which enabled us to avoid exposure recall bias. We selected subjects from the general population, we kept the sample size large, the response rate for the surveys was acceptable given its setting, and the loss to follow-up was negligible. In addition, the registries of cancer, stroke, and myocardial infarction were of sufficient quality to reduce the misclassification of outcomes. To our knowledge, this is the first prospective cohort study to examine associations between vitamin supplement use pattern and risk of cancer and CVD incidence simultaneously.

## Conclusions

Allowing for the methodologic issues, our results from a population-based prospective cohort study in Japan suggest that vitamin supplement use pattern has an impact on the subsequent risk of total cancer and CVD in women but not men. Elevated risk of cancer among women who were past and recent users of vitamin supplements may be partly explained by preexisting diseases or unhealthy background, which could not be completely controlled for in our study. Although consistent use of vitamin supplements for women might possibly reduce the risk of CVD, further research with detailed long-term data regarding components, doses, and patterns of vitamin supplement use is needed to confirm the generalizability of our findings.

## Competing interests

The authors declare that they have no competing interests.

## Authors' contributions

We thank all staff members in each study area for their painstaking efforts to conduct the survey and follow-up. The authors' responsibilities were as follows: ST (principal investigator); M. Inoue, conducted the study, managed the cancer data collection; HI, managed the CVD data collection; AH, analyzed and interpreted the data and prepared the manuscript; SS, M Iwasaki, TS, NS, TY, and JI helped to conduct the study. All authors provided critical suggestions for revision of the manuscript. All authors read and approved the final manuscript. AH received a research resident fellowship from the Foundation for Promotion of Cancer Research (Japan) for the 3rd term Comprehensive 10-year Strategy for Cancer Control.

## Pre-publication history

The pre-publication history for this paper can be accessed here:

http://www.biomedcentral.com/1471-2458/11/540/prepub
